# Role of EZH2 in Epithelial Ovarian Cancer: From Biological Insights to Therapeutic Target

**DOI:** 10.3389/fonc.2013.00047

**Published:** 2013-03-13

**Authors:** Hua Li, Rugang Zhang

**Affiliations:** ^1^Gene Expression and Regulation Program, The Wistar InstitutePhiladelphia, PA, USA

**Keywords:** epithelial ovarian cancer, EZH2, PRC2, EZH2 inhibitors, epigenetic therapeutics

## Abstract

EZH2 is the catalytic subunit of polycomb repressive complex 2 (PRC2), which generates a methylation epigenetic mark at lysine 27 residue of histone H3 (H3K27me3) to silence gene expression. EZH2 target genes are involved in a variety of biological processes such as stem cell pluripotency, cell proliferation, and oncogenic transformation. EZH2 is often over-expressed in epithelial ovarian cancer (EOC) cells and in ovarian cancer-associated stromal endothelial cells. Notably, EZH2 promotes cell proliferation, inhibits apoptosis and enhances angiogenesis in EOCs. In contrast to genetic alterations, which are typically non-reversible, epigenetic alterations are reversible. Thus, inhibiting EZH2/PRC2 activity represents an attractive strategy for developing ovarian cancer therapeutics by targeting both ovarian cancer cells and ovarian tumor microenvironment. Here we discuss the progress recently obtained in understanding how EZH2/PRC2 promotes malignant phenotypes of EOC. In addition, we focus on strategies for targeting EZH2/PRC2 to develop novel EOC epigenetic therapeutics.

Epithelial ovarian cancer (EOC) accounts for more death than any other gynecological malignancies in the developed world (American Cancer Society, 2012). Thus, there is an urgent need to understand the etiology and biology of the disease to develop new therapeutics. EZH2 catalyzes lysine 27 methylation on histone H3 to epigenetically silence the expression of its target genes (Bracken et al., 2006). EZH2 by itself is catalytically inactive and has to complex with other components of polycomb repressive complex 2 (PRC2) to exert its enzymatic activity (Cao and Zhang, 2004; Ketel et al., 2005). At minimum, it requires SUZ12 and EED subunits, while RbAp48 subunit can further boost the methyltransferase activity of EZH2 (Cao and Zhang, 2004). PRC2/EZH2 target genes are highly enriched for pathways that regulate stem cell pluripotency, cell proliferation, and oncogenic transformation (Kleer et al., 2003; Ting et al., 2006; Jones and Baylin, 2007; Schwartz and Pirrotta, 2007; Jaenisch and Young, 2008; Li et al., 2012a). Not surprisingly, components of PRC2 are often over-expressed in human cancers and their expression often positively correlates with aggressiveness in these diseases (Varambally et al., 2002; Kleer et al., 2003; Li et al., 2010, 2012b). In addition, gain-of-function mutations in EZH2 have also been reported in certain hematopoietic malignancies (Ryan et al., 2011; Majer et al., 2012). Notably, it has been shown that EZH2 and other components of PRC2 play a key role in regulating proliferation, apoptosis, and invasion of human EOC cells (Li et al., 2010, 2012b; Lu et al., 2010). In addition, EZH2 regulates EOC microenvironment by enhancing angiogenesis (Lu et al., 2010). Significantly, highly specific small molecule inhibitors of EZH2 methyltransferase activity have recently been developed (Knutson et al., 2012; McCabe et al., 2012b; Qi et al., 2012). Here we will discuss latest advances in understanding the biological function and regulation of EZH2/PRC2 in EOC as well as the therapeutic targeting EZH2/PCR2 in this devastating disease.

## Expression and Regulation of EZH2/PRC2

EZH2, when presents in PRC2, can add up to three methyl groups to the lysine 27 of histone H3 (H3K27Me3) via its SET domain methyltransferase (Bracken et al., 2006). EZH2 is often over-expressed in human EOC cells compared with normal human ovarian surface epithelial cell (Li et al., 2010). In addition, there is recent evidence to suggest that a proportion of high-grade serous EOC may arise from distal fallopian tube epithelial cells (Levanon et al., 2008; Kurman and Shih, 2010; Kuhn et al., 2012). Notably, compared with normal fallopian tube epithelium, EZH2 is over-expressed in high-grade serous EOC (Li et al., 2012b). Likewise, other components of PRC2 such as SUZ12 are also over-expressed in EOC compared with either normal human ovarian surface epithelial cells or fallopian tube epithelial cells (Li et al., 2012b). Indeed, the expression of EZH2 and SUZ12 positively correlates with each other in human EOCs, further supporting the idea that EZH2 functions within PRC2 in EOC (Li et al., 2012b).

Interestingly, although EZH2 upregulation correlates with an increased level of H3K27Me3 in EOC cell lines (Li et al., 2010), there is evidence to suggest that EZH2 levels do not correlate with H3K27Me3 levels in primary EOC specimens (Wei et al., 2008). There are a number of possible reasons for this phenomenon. First, although the core subunits of PRC2 such as EZH2, SUZ12, and EED are over-expressed in EOC cells (Li et al., 2010, 2012b), the expression of other PRC2 associated proteins such as RbAp48 and AEBP2 can also affect the methyltransferase activity of EZH2 and/or the recruitment of PRC2 to its target genes (Cao and Zhang, 2004). For example, *in vitro* histone methyltransferase assay showed that addition of RbAp48 to EZH2–EED–SUZ12 complex or AEBP2 to the EZH2–EED–SUZ12–RbAp48 complex significantly increased the methyltransferase activity of PRC2 (Cao and Zhang, 2004). In addition, it was recently found that polycomb-like (PCL) family proteins such as PHF19 play a critical role in the recruitment of PRC2/EZH2 to its target genes (Ballare et al., 2012). Biochemically, C-terminal end of PHF19 binds to SUZ12 in the context of an intact PRC2 (Ballare et al., 2012). Knockdown of PHF19 did not affect the stability of the PRC2 complex, but the association of PRC2 with the promoters of target genes was substantially reduced and, consequently, vast majority of target genes lost their H3K27Me3 epigenetic mark (Ballare et al., 2012). Likewise, JARID2 is also important for the recruitment of PRC2 to its target genes (Landeira et al., 2010). Together, these findings demonstrated that a stable association of PRC2 at target genes is required for its gene silencing function and PRC2 associated proteins play a key role in regulating the recruitment of PRC2 to its target genes. Another class of regulators of PRC2’s genomic localization is non-coding RNAs such as HOTAIR (Tsai et al., 2010), XIST (Zhao et al., 2008), and intronic RNAs (Guil et al., 2012). They bind directly to PRC2 to regulate its association with target genes. Finally, histone demethylases such as UTX and JMJD3 can decrease the levels of H3K27Me3 (Agger et al., 2007) and loss of function mutations in UTX have been reported in human cancers (Gui et al., 2011; Jankowska et al., 2011). Thus, it will be interesting to examine the correlation between expression of core subunits of PRC2 and expression of PRC2 associated proteins, non-coding RNAs and histone demethylases in EOC.

In addition to over-expression, gain-of-function EZH2 mutations have been reported in hematopoietic malignancies (Ryan et al., 2011; Majer et al., 2012). The most common mutations occur within the SET domain that confers methyltransferase activity. For example, Y641 mutations have been identified in ∼20% of germinal-center diffuse large B-cell lymphomas and in ∼7% of follicular lymphomas (Bodor et al., 2011). Interestingly, these mutations are always heterozygous and the resulting cells also carries a wild type copy of EZH2 (Morin et al., 2010; Sneeringer et al., 2010). Mechanistically, the mutant EZH2 exhibits little to no activity for H3K27Me0 substrate and a decreased activity for H3K27Me1 substrate, while display significantly increased activity for H3K27Me2 substrate (Sneeringer et al., 2010). Consequently, the wild type EZH2 retained in these cells accounts for the generation of H3K27Me1 and H2K27Me2, which led to a global increase in H3K27Me3 by the mutant EZH2 in these tumor cells. Notably, A677 mutations in the EZH2’s SET domain also occur in human lymphoma albeit at much lower rate compared with Y641, which exhibits an increased enzymatic activity independent of H3K27 methylation status (McCabe et al., 2012a). This led to a global increase in H3K27Me3 levels (McCabe et al., 2012a). Regardless, EZH2 mutation has not been reported in EOC and there is no evidence of EZH2 mutation in EOCs based on the newly released The Cancer Genome Atlas (TCGA) EOC database.

In response to intrinsic and extrinsic cues, EZH2 activity is regulated by post-translation modification such as phosphorylation. For example, T350 residue of EZH2 is subject to cyclin-dependent kinases (CDK) 1 and 2 during S and G2/M phases of the cell cycle, respectively (Chen et al., 2010). T350 phosphorylation enhances PRC2/EZH2-mediated gene silencing through increasing the recruitment of PRC2 to its target genes, while has no effect on PRC2 complex assembly or EZH2 enzymatic activity (Chen et al., 2010). Thus, this mechanism links intrinsic cell cycle progression to EZH2 function. In addition, extracellular cues can also affect EZH2 function by signaling activated kinases such as AKT (Cha et al., 2005) and p38 (Palacios et al., 2010). S21 residue of EZH2 can be phosphorylated by AKT, which decreases histone methylation by PRC2 (Cha et al., 2005). Interestingly, recent evidence suggests that phosphorylation of EZH2 by AKT at S21 site leads to PRC2-independent association of EZH2 with androgen receptor and activation of its target genes in prostate cancer cells (Xu et al., 2012). Given that AKT is often hyperactive in EOC (Altomare et al., 2004), it is possible that AKT directly regulates EZH2 in a PRC2-independent manner to activate certain genes in EOC. In addition, in skeletal muscle stem cells, stress response kinase p38 alpha phosphorylates T372 residue of EZH2 in response to exposure to TNF inflammatory cytokine (Palacios et al., 2010). This leads to an enhanced repression of PRC2 target skeletal muscle stem cell marker to promote cell differentiation. Notably, the relevance of these post-translation phosphorylation modifications in EOC remains to be determined.

## Role of EZH2/PRC2 in EOC

Components of PRC2 are often over-expressed in human EOC cells and their expression positively correlates with markers of cell proliferation such as Ki67 (Li et al., 2010, 2012b). Significantly, higher levels of EZH2 (Lu et al., 2010) or SUZ12 (Li et al., 2012b) expression predict shorter overall survival in EOC patients. Indeed, knockdown of EZH2 (Li et al., 2010) or SUZ12 (Li et al., 2012b) suppresses the growth of human EOC cells *in vitro* and *in vivo* in both subcutaneous and orthotopic xenograft EOC models in immunocompromised mice. This is due to induction of programed cell death or apoptosis (Li et al., 2010, 2012b). In addition, knockdown of EZH2 suppresses the invasion of human EOC cells (Li et al., 2010; Lu et al., 2010; Rao et al., 2010), which correlates with a decrease in transforming growth factor-beta 1 (TGF β1) expression and an increase in E-cadherin expression (Rao et al., 2010). These observed phenotypes correlate with a decrease in the levels of H3K27Me3 in these cells (Rao et al., 2010). Together, these findings are consistent with the notion that EZH2/PRC2 promotes EOC by suppressing cell apoptosis (Li et al., 2010, 2012b) and increasing their invasion potential (Li et al., 2010; Rao et al., 2010) by silencing its target genes through H3K27Me3 epigenetic mark.

Notably, EZH2 expression is upregulated in ovarian cancer stem cell-like cells enriched by chemotherapy (Rizzo et al., 2011) and knockdown of EZH2 leads to loss of stem cell-like properties in these cells (Rizzo et al., 2011), such as anchorage-independent growth and tumor growth in xenograft mouse model. Similarly, it has been demonstrated that compared with cisplatin sensitive parental EOC cells, EZH2 expression is upregulated in *in vitro* derived cisplatin-resistant EOC cells (Hu et al., 2010). Conversely, knockdown of EZH2 re-sensitized drug-resistant ovarian cancer cells to cisplatin (Hu et al., 2010). Together, these reports support the idea that EZH2 may contribute to chemoresistance by regulating stem-like cell population in EOCs.

Interestingly, EZH2 is also over-expressed in EOC-associated endothelial cells due to a direct paracrine VEGF stimulation (Lu et al., 2010) and EZH2 promotes angiogenesis by silencing vasohibin1 (VASH1) (Lu et al., 2010). Consistently, EZH2 knockdown in the EOC-associated endothelial cells inhibits angiogenesis and reduces EOC growth, which is further enhanced by EZH2 knockdown in EOC cells (Lu et al., 2010). Thus, EZH2 also contributes to malignant behaviors of EOC by altering EOC-associated tumor microenvironment through promoting angiogenesis. Notably, recent anti-angiogenesis clinical trials by targeting VEGF demonstrated potential benefit when added to standard chemotherapy in the first-line treatment of ovarian cancer (Burger et al., 2011; Perren et al., 2011). Together, these observations suggest that EZH2 is a potential target for designing new anti-angiogenesis therapy in EOC.

Genome-wide chromatin immunoprecipitation followed by deep sequencing analysis and gene expression profile reveal that the number of genes upregulated upon EZH2 knockdown is significantly lower than the number of genes whose genomic loci are directly occupied by EZH2/H3K27Me3 (Li et al., 2012a). This is consistent with the observation that histone deacetylase (Van der Vlag and Otte, 1999) and DNA methylation (Nakamura et al., 2008) also regulate the expression of EZH2 target genes. In addition to VASH1, the cellular networks enriched by EZH2 target genes in EOC cells include cell death, growth and proliferation and reproductive system development, and cancer (Li et al., 2012a). These findings further support EZH2’s proliferation-promoting and apoptosis-suppressing function. Notably, Harakiri (HRK), a proapoptotic gene, is subjected to H3K27Me3 mediated gene silencing in EOC cells and HRK plays a key role in regulating apoptosis induced by suppressing H3K27Me3 (Li et al., 2012b). HRK is also subjected to DNA methylation (Nakamura et al., 2008) mediated gene silencing, further suggesting possible cooperation between H3K27Me3 and DNA methylation mediated gene silencing.

## Therapeutic Targeting EZH2/PRC2 in EOC

In contrast to genetic changes that are non-reversible, epigenetic alterations are reversible. This feature makes epigenetic alterations in cancer an ideal target for developing cancer therapeutics (Dawson and Kouzarides, 2012). Significantly, EZH2 is over-expressed in both EOC cells and EOC-associated endothelial cells and its knockdown suppresses EOC growth (Li et al., 2010; Lu et al., 2010). These observed effects correlate with a decrease in levels of H3K27Me3, the enzymatic product of EZH2 methyltransferase (Li et al., 2010; Lu et al., 2010). Together, these findings strongly suggest that EZH2’s methyltransferase activity is a potential target for developing urgently needed EOC therapeutics.

The first reported inhibitor of EZH2’s methyltransferase activity is DZNep, an inhibitor of *S*-adenosylhomocysteine (SAH) hydrolase (Tan et al., 2007). However, DZNep indirectly inhibits EZH2 activity by causing the degradation of components of PRC2 through increasing the cellular levels of SAH, the inhibitory byproduct of methyltransferase reaction (Tan et al., 2007). In addition, DZNep is non-specific with major effects on a number of other histone methyl marks and, consequently, has been shown to be very toxic (Miranda et al., 2009). In contrast, a number of new specific EZH2 methyltransferase inhibitors have been reported very recently including most notably GSK126 (McCabe et al., 2012b) and EPZ005687 (Knutson et al., 2012) and EI1 (Qi et al., 2012). These inhibitors act in a *S*-adenosylmethionine (SAM) competitive manner with no effects on expression of components of PRC2 or other histone methyl marks. This characteristic of the newly developed inhibitors allows to distinguish the EZH2’s methyltransferase-dependent and -independent function. In a panel of B-cell lymphoma cell lines, EZH2 methyltransferase specific inhibitors are particularly sensitive in cells harboring mutations in Y641 or A677 residue (McCabe et al., 2012b). Interestingly, the cell line with A677 mutation appears to be most sensitive, which correlates with the fact that A677 mutant efficiently catalyzes all three steps of H3K27 methylation while Y641 mutant preferentially enhance the reaction from di- to trimethylation (McCabe et al., 2012a). In addition, it appears that loss-of-function mutation in H3K27Me3 demethylase UTX may also confer sensitivity to EZH2 inhibitors (McCabe et al., 2012b). However, gain-of-function mutation is not sufficient to predict response to EZH2 inhibitors because certain cell lines with EZH2 mutation are not sensitive to EZH2 inhibitors (McCabe et al., 2012b). Significantly, EZH2 inhibitor GSK126 eradicated the growth of xenografted B-cell lymphoma cells with EZH2 mutations (McCabe et al., 2012b). Despite the fact that EZH2 is essential during development and is known to regulate normal tissue stem cell properties, mice tolerated GSK126 very well with no apparent toxicity (McCabe et al., 2012b). Together, these findings suggest that EZH2 inhibitors such as GSK126 are potential novel epigenetic intervention reagents for B-cell lymphoma harboring gain-of-function EZH2 mutations.

EZH2 inhibitors are equally effective in decreasing H3K27Me3 in cancer cell lines with wild type EZH2 compared with those with gain-of-function EZH2 mutations (Knutson et al., 2012; McCabe et al., 2012b). However, cell lines with wild type EZH2 are typically much less sensitive to GSK126 (McCabe et al., 2012b). This observation suggests that methyltransferase-independent function of EZH2 also contributes to the malignant phenotypes observed in EZH2 over-expressing cancer cells. Notably, EZH2 is over-expressed but not mutated in EOC. In addition, inhibition of PRC2 by knockdown EZH2 (Li et al., 2010) or SUZ12 (Li et al., 2012b) is effective in inhibiting the growth of EOC cells with over-expressed components of PRC2. Therefore, it will be critical to evaluate the effectiveness of EZH2 inhibitors in preclinical models for their effects on EOC cell growth as well as EOC-associated angiogenesis. In addition to inhibiting EZH2’s methyltransferase activity, targeting the PRC2 complex formation, or the associated factors that affect PRC2 genome-wide distribution may represent an alternative strategy for targeting PRC2 in EOCs. Further, given the evidence that EZH2 target genes may also subject to other epigenetic silencing mechanisms (Van der Vlag and Otte, 1999; Nakamura et al., 2008), it will be interesting to examine whether there are synergies between EZH2 inhibitors and inhibitors of other epigenetic silencers such as HDAC and DNA methyltransferase inhibitors. Finally, given its potential role in conferring chemotherapy resistance (Knutson et al., 2012; McCabe et al., 2012b; Qi et al., 2012), it will be interesting to determine whether EZH2 inhibitors re-sensitize chemo-resistant EOC cells.

## Conclusion and Perspective

In summary, EZH2 and other components of PRC2 are often over-expressed in EOC cells (Li et al., 2010, 2012b) and EOC-associated endothelial cells (Lu et al., 2010). EZH2/PRC2 promotes proliferation, suppresses apoptosis and increases invasion potential of EOC cells (Li et al., 2010), and enhances angiogenesis of EOC-associated endothelial cells (Lu et al., 2010). The expression pattern and functional importance of EZH2/PRC2 establish it as an exciting target for developing new EOC therapeutics. Thus, it will very interesting to test the newly developed EZH2 inhibitors in relevant preclinical *in vitro* and *in vivo* models. In addition to H3K27 residue, EZH2 can also methylate other proteins. For example, EZH2 methylates an orphan nuclear receptor RORa (Lee et al., 2012). Further, EZH2/PRC2 may also have methyltransferase-independent function. Indeed, EZH2 is present in both cytoplasm and nuclei and cytoplasmic EZH2 has been shown to regulate actin polymerization and cell signaling (Su et al., 2005). Accordingly, a further understanding the mechanisms by which EZH2/PRC2 promote EOC will provide scientific rationale for developing inhibitors of EZH2/PRC2 as novel EOC therapeutics.

## Conflict of Interest Statement

The authors declare that the research was conducted in the absence of any commercial or financial relationships that could be construed as a potential conflict of interest.
